# Environmental and Behavioral Factors in Association with Lung Function Impairment in Children Living in Wuhan, China

**DOI:** 10.3390/ijerph20021134

**Published:** 2023-01-09

**Authors:** Suzhen Cao, Sai Li, Xiaoli Duan, Yaqun Zhang, Jicheng Gong, Qian Guo, Xiangyu Xu, Li Peng, Xin Meng

**Affiliations:** 1 Beijing Key Laboratory of Resource-Oriented Treatment of Industrial Pollutants, University of Science and Technology Beijing, Beijing 100083, China; 2 Gansu Provincial Design and Research Institute of Environmental Science, Lanzhou 730000, China; 3 College of Environmental Science and Engineering, Peking University, Beijing 100871, China; 4 Chinese Academy of Environmental Planning, Ministry of Ecology and Environment of the People’s Republic of China, Beijing 100043, China

**Keywords:** lung function, school children, household characteristics, parental factor, socioeconomic levels

## Abstract

Children’s lung function is a significant predictor of health status throughout their lifetime. This study aims to identify the prevalence of impaired lung function in children and the potential influencing factors in elementary school children of Wuhan, China. Children of 6–12 years old were enrolled from elementary schools in Wuhan, China, in 2018, on the basis of a cross-sectional study design. Information on personal behavior patterns and household characteristics, as well as parental factors, was collected based on a questionnaire survey. Spirometry was used to measure fifteen lung function indicators. Logistic regression models were used to estimate odds ratios of prevalence of impaired lung function with regard to socioeconomic, personal behavior patterns, household, and parental factors, respectively. Four lung function indicators such as FET and EVC in urban children had higher values than in the suburban children after adjusting for confounders (*p* < 0.05). A higher prevalence of impaired lung function (FEV_6_, FEV_3_, EVC, and VC) was found in the children from the rural area than in those from the urban area. A sex difference in FET impairment was observed, however, no significant difference in impairment in other lung function indicators were found between girls and boys. The elevated height and weight of the children was significantly associated with an increased and decreased prevalence of impaired lung function, respectively, particularly both for FEV_6_, FEV_3_, FIVC, and FIV_1_. Opening windows for a longer time in summer was significantly associated with a lower prevalence of impaired FEF_25_ and MVV, and an extended time of opening windows in winter was significantly associated with a lower prevalence of impaired FEV_6_. While, opening windows for a longer time in autumn was significantly associated with higher prevalence of impaired FEV_6_ and FEV_1_/VC. Home renovations, doing physical exercise for more than 30 min per day, air pollution exposure during commuting, sleeping in own rooms, height stunting, and preterm birth were significantly associated with an increased risk of impaired lung function. Breastfeeding, having a father with a white-collar profession and with a higher education level were positively associated with the lower prevalence of impaired lung function. Impaired lung function is commonly found in school children in Wuhan, nowadays. Breastfeeding, opening windows long-term in summer and winter, higher socioeconomics, and an urban living environment were protective factors for impaired lung function. However, opening windows long-term and using air conditioning short-term in autumn, as well as home renovations, doing physical exercise for more than 30 min per day, preterm birth, height stunting, and air pollution exposure during commuting were regarded as significant risk factors for impaired lung function. Promoting breastfeeding, lengthening window opening times in winter and summer, and controlling household renovation and air pollution exposure during commuting are recommended to reduce the risk of impaired lung function in children in Wuhan.

## 1. Introduction

Lung function is regarded as an important indicator in the prevention and diagnosis of respiratory disease [1,2]. Aggravated lung function impairment is considered to be an important indicator of respiratory, cardiovascular, and all-cause mortality [3]. Some scholars have stated that a decrease in force expiratory volume in 1 s (FEV_1_) is an independent predictor of mortalities [4,5]. Low forced vital capacity (FVC) is evidenced as an important indicator of mortality in the general population [6]. Additionally, impairment in lung function is also related to type 2 diabetes [7,8] and cardiovascular diseases [9,10]. Furthermore, impaired lung function in childhood can lead to abnormal growth and development of adult lung function [11]. Thus, lung function measurement is commonly used as a tool in general health assessment.

It is well evidenced that lung function can be influenced by genetic factors [12], and non-genetic factors which are related to nutritional status, personal behavior activities, and socioeconomic and environmental factors [13,14,15]. A study conducted in Lanzhou, China, reported that long-term PM_2.5_ exposure was associated with a reduction in FVC and FEV_1_ in elementary-school age children [16]. A cross-sectional study focusing on the relationship between children’s respiratory health and the indoor environment and lifestyle in Chongqing, China, has demonstrated that frequent use of air fresheners is related to a reduction of lung function, while, high frequency consumption of dairy products, fruits, and vegetables, as well as daily exercise for more than 1 h, are to the benefits of lung development [17]. The influences of urban environments on lung function in children have also been assessed by considering various factors [18,19]. The effects of low socio-economic status on the impairment of children’s lung function have been studied, most of which were conducted in Europe or America [18,20].

Although the influencing factors which may lead to a decline in the development of children’ lung function has been widely studied based on follow-up or cross-sectional study designs [16,17,18,19,20,21], the evidence of effects of certain determinants, such as household and personal behavior activities, for example household ventilation condition, home renovation, physical activities, and environmental tobacco smoking exposure, etc., on the impairment of children’s lung function are still under-studied [22,23]. However, considering the differences in environmental conditions and lifestyles with different socioeconomic levels, the risk factors would be inconsistent in different areas. For example, He et al. reported that the growth rate of lung function is lower in urban children than rural children [18], while Renzetti et al. found a reversible association between urban environment and children’s lung function [19]. Thus, figuring out the critical factors that influence the impairment of children’s lung function is crucial in respiratory health protection of children.

Two decades ago, a panel study was carried out to test the lung function condition in children and to survey the detrimental risk factors in Wuhan, China [24,25]. To provide more targeted advice and scientific basis for the improvement of children’s respiratory health presently, we carried out a follow-up study with the same age range and same living areas in 2018. In the study, we propose to investigate the prevalence of impairment in lung function in children in Wuhan, China, and to explore the potential risk factors of the impaired lung function in recent years. 

## 2. Methods

### 2.1. Study Site and Participants

The description of the study site, i.e., Wuhan, China, has been detailed in the previous study [21]. In short, 9–12 years old children from two elementary schools in urban areas and from one elementary school in a rural area were recruited in 2018. After obtaining permission from the school administrators, a description of the study and a blank informed consent form which informed the voluntary and noncompulsory nature of their participation and was used to obtain the understanding and permission for the survey from the parents or guardians of the subjects, were handed out to the potential subjects. After collecting the signed consent forms, we conducted the questionnaire survey and lung function measurements in children. Finally, a total of 3152 children (male: 57.0%; female: 43.0%) took part in the questionnaire survey, and 383 children were enrolled in the lung function test with a distribution of male 219 (57.18%) and female 164 (42.82%). For the consistency of the participants, we finally included 383 children in the study who participated both in the questionnaire survey and lung function measurement. The study protocol was reviewed and approved by the Ethics Committee of Biomedical Research, Duke Kunshan University, Jiangsu (No. FWA00021580).

### 2.2. Questionnaire Survey and Data Collection

A questionnaire designed by the American Thoracic Society Epidemiologic Standardization Project was applied [26]. On this basis, we supplemented some questions which were related to personal activity patterns (e.g., doing physical exercise) and some emerging household influencing factors (e.g., presence of mold). The questions in the questionnaire were classified into the following categories: (I) children’s sociodemographic characteristics, such as gender, age, and location; (II) personal activity patterns, such as the frequency and type of physical exercise, whether they have environmental tobacco smoking (ETS) exposure, and dietary habits, including intake frequency of fruit, vegetable, seafood, dairy, and high-calories food; (III) parental health condition, such as whether their parents had doctor-diagnosed asthma and bronchitis; (IV) parental socioeconomic status, like parental occupation and education attainment; (V) children’s early life factors, such as whether a child was born prematurely, whether a child was breastfed, and the duration of exclusive breastfeeding; (VI) household living conditions, i.e., fuel types used for heating and cooking, kitchen ventilation devices, dampness and mold, renovation, air purifiers use, mosquito-repellent and incense stick use, air freshener use, ventilation duration in different seasons, and duration of air condition use in different seasons. 

### 2.3. Anthropometric Measurement

Children’s weight and height were measured with an anthropometer (Jiangsu Suhong Medical Equipment Co. Ltd., Jiangsu, China), with a respective minimum measurement of 0.1 kg and 0.5 cm in bare feet. Body mass index (BMI) (kg/m^2^) was obtained using the weight (kg) divided by height (m) squared. The WHO 2006 definition [27] and National Health Commission of the People’s Republic of China on standard for height level classification [28] were used as the evaluation criteria for stunting in children. Stunting (z-scores < −2SD) and no stunting (z-scores ≥ −2SD) were assessed based on the height values for sex and age [29]. The screening method for obesity and overweight among school-age children and adolescents from The National Health Commission of the People’s Republic of China [30] was used as the evaluation criteria for obesity and overweight in children.

### 2.4. Measurement of Lung Function

All lung function tests were performed as recommended [31], by trained technicians using a portable spirometer Ⅲ (Spiro-lab MIR, co. Ltd., Rome, Italy). In each school, children were required to take the lung function measurement in a standing position in a dedicated room during school hours. To our best knowledge, the parameters such as FEV_1_ and FVC were usually measured in most studies, but little information on the other parameters such as vital capacity (VC) and maximal voluntary ventilation (MVV) can be obtained, which is not good for the overall understanding of lung function status. Thus, fifteen other lung function indexes, such as VC, MVV, FEF_25_, FEV_3_, and FEV_6_, etc., were measured in this study. The spirometry test was repeated (up to five times) until presenting acceptable, reproducible flow-volume loops were obtained for each participant [32]. Between one test and the next, the device evaluates the repeatability by the following parameters: repeatable when the difference between the two highest forced vital capacity values was ≤150 mL and the forced expiratory volume in 1 s value was ≤150 mL [32]. Acceptable spirograms were defined as smooth flow-volume curve without artifacts, and satisfactory exhalation with a forced expiratory duration > 6 s [32]. If the above principles were not met, the monitoring was considered invalid and re-measurement was required. If the difference between the two largest forced vital capacity values was within 150 mL, the test was concluded. The two largest acceptable and concluded values of the parameters were selected and averaged for statistical analysis.

### 2.5. Definition of Impaired Lung Function and Influence Factors

The Global Lung Initiative (GLI) 2012 equations were used to determine reference lung volumes for each indicator to compute the percentage of the predicted index [33]. Reduced lung function was defined as the measured value of index was lower than 80% of the GLI predicted reference value. The rate of reduction for each index was then calculated [34,35]. The socioeconomic class of the participants was determined based on the parental highest educational level and occupation [36]. Kitchen style refers to an enclosed kitchen (which was not separated to living room) or a separated kitchen (see Figure S1), coal fuel use was defined from the question of “what was the main fuel type for cooking?” and “what was the main fuel type for heating?” Parental education level was set as a binary variable: below college vs. with or above college. Parental occupation was classified by other occupations without a fixed income, blue collar (e.g., farmers and factory workers), and white collar (e.g., teachers and governmental office workers). Environmental tobacco smoking (ETS) exposure was considered present if there was any smoker living with the child. 

Doing physical exercise was assessed by the “yes” answer to the questions of “do you do physical exercise in school?” and “do you do physical exercise out of school?”. Ambient air exposure during commuting was decided by the question of “what was the main way of transportation to and from school?”. The open mode of transportation (on foot, bicycle, and motorbike) was collapsed to create a binary variable: air pollution exposure during commuting (yes vs. no). As for dietary habits, we investigated separately for each type. Various diet behaviors were obtained from the question of “what was the frequency of taking this food?”. The frequency of taking vegetables and taking dairy, etc., was divided into certain times per day or certain times per week. 

### 2.6. Statistic Analysis

Data was tested for homogeneity (Bartlett’s test for unequal variances) and normality (Shapiro-Wilks W-test). Continuous variables (e.g., age) were expressed as mean ± standard deviation (SD) and categorical variables (e.g., gender) were presented as the frequency (percentage) in each subgroup. Discrepancy in lung function between categorical variables (e.g., district) were analyzed using a Wilcox test, whereas differences in the prevalence of impairment in lung function between two areas were analyzed by Pearson’s Chi-square (χ2) tests. The association between variables and the prevalence of impaired lung function was examined by univariate logistic regression analyses. Adjusted odds ratios (OR) with 95% confidence intervals (95% CI) were analyzed for every independent variable. These variables included sex, age, weight, height, district, ETS exposure, sleeping in their own room, kitchen type, ventilation use when cooking, household coal use, parental education level, and parental occupation, etc. Statistical significance was declared when a *p* value was <0.05 and the statistical tests were two tailed. All analyses were conducted by SPSS software for Windows, version 22 (SPSS Inc., Chicago, IL, USA).

## 3. Results

### 3.1. Characteristics of Anthropometric, Parental, Household and Socioeconomic Factors

Details about the sociodemographic information and personal behavior patterns of the children are shown in Table 1. Children’s mean (SD) age was 9.4 (1.2). More urban (63.4%) and male (57.2%) children were recruited. Children from the urban area were older, taller, and heavier than those from the rural area (*p* < 0.001) (Table S1). A larger fraction of the children were breastfed during infancy (81.1%). A portion of 8.6% were preterm born among the children. Of the children, 73.2% and 83.0% did physical exercise and experienced ETS exposure, respectively. In comparison with seafood and high-calorie food consumption, more than 80% of the children ate vegetables, fruit, and dairy 1–3 times in each day. A higher fraction of the children (77.0%) had air pollution exposure during commuting to school. Most children had their own beds and own bedrooms. The rates of higher education levels (with or above college) in mothers and fathers were both more than 30%. The children’s mother and father were observed as being white-collar workers at significant higher rates. Only 2.9% of households were found to use coal in cooking and heating. Nearly 70% of households had an enclosed kitchen and used mosquito coil, while 21% of households used air purifiers. The duration of opening windows in spring to autumn was approximately 14 h per day, while the duration in winter was 9.2 h. The duration of air conditioning use in spring, summer, autumn, and winter were 0.7, 8.6, 0.6, and 2.1 h per day, respectively. In comparison with maternal smoking, a prevalence of 47.4% was found in paternal smoking. Parental asthma was found at a low rate and the prevalence of parental bronchitis was about 5% to 6%. 

### 3.2. Reduction of Children’s Lung Function

The values of the fifteen lung function indexes are shown in Table 2. Except for FEV_1_/VC, FEV_3_/FVC, and MVV, the other indexes, such as FET and FEV_6_, had a significantly higher value in urban children than suburban children (Table 2). However, after adjusting for confounders, only higher FET, EVC, IC, and VC were observed in urban children, the other lung function indexes in urban children were comparable to or lower than those in rural children. Table 3 shows the prevalence of reduced FEV_3_ and FEV_6_ was 34.7% and 46.7%, respectively. The unadjusted raw prevalence of reduced FEV_6_, FEV_3_, EVC, IC, and VC in suburban children was higher than in urban children. After controlling the confounding factors such as sex, age, weight, and height, as well as potential parental and household factors, FEV_6_, FEV_3_, EVC, and VC had a significantly higher prevalence of impairment in suburban area (*p* < 0.05 or *p* < 0.01). The prevalence of reduced lung function was comparable between males and females (see Table S2).

### 3.3. Effects of Household, Sociodemographic, and Parental Factors on Reduced Lung Function 

Table 4 shows the ORs of the prevalence of reduced lung function associated with household, sociodemographic, and parental factors. It shows that the children who were taller or lighter were both more likely to develop reduced lung function, while no association between gender, presence of pets, presence of mold, air purifier use, enclosed kitchen, coal fuel use, ventilation use during cooking, maternal education level, maternal bronchitis, EST exposure, and reduced lung function was found. A negative effect of suburban living environment was observed on lung function development. However, a positive effect of breastfeeding was found. Children from the families opening windows for a longer time in summer or in winter were associated with a significant lower prevalence of reduced lung function, for example, the adjusted ORs of FEF_25_ for the children whose family open windows for a longer time in summer and the adjusted ORs of FEV_6_ for the children whose family open windows for a longer time in winter were 0.905 (95% CI: 0.753, 0.975) and 0.908 (95% CI: 0.796, 0.978), respectively. However, opening windows for a longer time in autumn was significantly associated with reduced FEV_6_ (OR = 1.111, 95% CI: 1.108, 3.651) and FEV_1_/VC (OR = 1.249, 95% CI: 1.076, 1.450). Using air conditioning in summer for a longer time was significantly associated with reduced FEF_25_ (*p* < 0.05) and FEF_50_ (*p* < 0.05), while using air conditioning for a longer time in autumn was significantly associated with a lower prevalence of reduced FIVC (*p* < 0.05) and ERV (*p* < 0.05). A higher prevalence was observed in reduced FEV_6_ (OR = 2.786, 95% CI: 1.293, 6.003) for the children whose household had home renovation. Fathers being white collar and having a higher education level were significantly associated with lower risks of reduced lung function. However, children whose mothers were white collar had higher ORs in reduced FEF_25_ (OR = 3.650, 95% CI: 1.299, 10.257) and FEF_50_ (OR= 4.116, 95% CI: 1.522, 11.131). Sleeping in their own room was significantly associated with higher risks of reduced FIV_1_/FIVC (OR = 2.855, *p* < 0.05) and FEV_1_/VC (OR = 3.525, *p* < 0.01).

Doing physical exercise was significantly associated with a higher risk of reduced FEV_6_ (OR = 2.265, *p* < 0.05). Being born preterm showed a negative effect to the development of lung function, since higher ORs of reduced lung function, particularly for FEV_6_, FEV_3_ and FIVC, were observed for the children who were born preterm. Additionally, height stunting showed a significant association with reduced ERV (OR = 21.170, *p* < 0.05), and air pollution exposure during commuting to school was associated with a higher prevalence of reduced lung function, although there was no statistical significance.

## 4. Discussion

Lung function impairment is increasingly evidenced to have a greater contribution to several health issues, such as all-cause mortality [3], asthma [23], and death from cardiovascular disease [9,10]. Due to individual, behavioral, and environmental factors [13,14,15,16,17], children’s lung function impairment was largely found. A recent cross-sectional Danish multi-centre study has reported that the mean FEV_1_ was 46.1% of predicted values, although the prevalence of severely impaired lung function was about 13 per 100,000 individuals [37]. A study conducted in Mexico City from 1996 to 1999 has reported a decrease in lung function growth is associated with air pollution in 3170 children aged 8 to 11 years [38]. Coincidentally, the same finding was also observed in China from past to present. A cross-sectional study conducted 25 years ago, focusing the school-age children in Guangzhou, Lanzhou, Wuhan, and Chongqing, has found a decrease in FVC growth affected by PM_2.5_ and PM_10_ [25]. Our previous studies have found that lung function in children currently in Lanzhou [16] and Wuhan [21] was decreased in comparison with 25 years ago, due to air pollution and various other influence factors. A cross-sectional study conducted in seven Chinese cities reported a prevalence of impaired lung function ranging from 6.8% to 11.3% [22]. Additionally, many recent cohort studies have shown that significant impairment in lung structure and lung function remains a major concern [39,40]. In this study, we also found that most of the lung function indicators were lower than the predicted values, indicating approximately half of children had impaired lung growth. Furthermore, more than half of children had lower spirometric values in comparison with the corresponding predicted values, among the fifteen indicators. Thus, the reduction of lung function in children should be a concern both in the making of prevention measures and the identification of influence factors. 

We previously illustrated that taller children in Wuhan, Hubei, have great values in FVC and FEV_1_ [21], but we also observed a greater prevalence of impaired lung function in 6–12 years taller children in this study, since the ORs values of FEV_6_, FEV_3_, FIVC, and FIV_1_ were statistically larger than 1. As for weight, we observed that the children with higher weight would have higher lung function values both 25 years ago and at present [21]. While, an increasing weight was consistently associated with a lower risk of the reduction in children’s lung function, as the ORs values of FEV_6_, FEV_3_, FIVC, and FIV_1_ were within 1. This result provides evidence that most spirometric values reduce when weight is low [41], but is contrary to that of the simultaneous study conducted in Lanzhou, China, which has the same study design as this study [42]. These differences could be interpreted by the fact that single BMI was used to assess obesity in these studies, ignoring other involved factors such as waist-to-hip ratio and waist circumference. A study carried out in the United Kingdom found that lung function values increased with increasing BMI up to a marginal BMI of 23 [43]. Given the average BMI of 17.4 ± 3.1 kg/m^2^ in this study, our finding indicating lower impairment in lung function in heavier children is supported by the cohort study in Toronto [44]. Furthermore, in order to assess the effects of stratified BMI on the lung function development in children, we compared the lung function levels and the prevalence of impaired lung function in children based on different BMI, i.e., normal, overweight, and obese, as shown in Table S3. We found there were significant differences in the children’s lung function indicators among the children with different BMI conditions and found that the overweight and obese children had higher levels of lung function, such as FEV_3_ and FEF_25_, and a lower prevalence of impaired lung function. However, the association of stratified BMI to children’s impaired lung function after controlling other confounders indicates that the higher prevalence of impaired FEV_6_, FEV_3_, FEF_25_, FIVC, and FIV_1_ were found in overweight children (Table S4); the prevalence of impairment in other lung function indexes was comparable among the children with different BMI.

We found a detrimental effect of suburban living environment on the prevalence of reduced lung function among elementary school children in Wuhan, Hubei, after adjusting for weight, height, household, and parental factors. In our other studies, a statistical association between living in a suburban area and lower lung function was observed in children from Wuhan and Lanzhou, China [21,42]. Thus, we further concluded that living in suburban areas would not only lead to a lower lung function, but also to a higher prevalence of impaired lung function in our study area. However, some other studies reported contrary results. A cohort study carried out in Canada has illustrated that higher lung function was observed among 6–14 years children living on a farm because of a “farm effect” [45]. Since the influence of the living environment on children’s lung function could be primarily originated from various critical factors, like environmental pollution [13,16], socioeconomic status [18,20], and household conditions [19,21], we need to further analyze these factors behind the urban or rural effects on lung function development. In this study, as a rapidly developing suburb, i.e., Huangpi district which was detailed in Ref. [21], large amounts of atmospheric pollutants were emitted. Given that the heavy air pollution in Wuhan has a tendency to transfer from urban to rural areas, and there is no clear differentiation or border between suburban and urban areas in the determinant household factors, including ventilation condition, and cooking/heating fuels [21], we speculated that the heavy air pollution and lower socioeconomic levels in this suburban area may increase the risk of impairment in children’s lung function, since air pollution was demonstrated as a critical detrimental factor to lung function growth and higher socioeconomic levels were conducive to lung function development [18,20]. Additionally, breastfed children had a lower prevalence of impaired lung function than non-breastfed children, although no stable association between duration of breastfeeding and risk intensity of impaired lung function was found for some indicators. This result ascertains the finding that breastfeeding has an increasing beneficial effect on lung function development in school-aged children in nowadays Wuhan [21]. Though we paid no attention to whether a child was exclusively breastfed, we established that approximately 64.3% of children were breastfed more than 4 months. Since breast milk is rich in immune factors, it may be more effective in promoting lung immune development and maturation than formula feeding or early consumption of solid food [46]. A cross-sectional study involved with elementary and middle schools distributed in seven cities in China [22] supported our fore-mentioned positive effects of breastfeeding on lung function.

The association between household renovation exposure and impaired lung function has already been reported on a limited basis and suggests that household renovation has a detrimental effect on lower lung function and impaired lung function [47,48]. A study conducted in seven Chinese northeastern cities demonstrated statistically significant differences in the prevalence of impaired lung function between children without and with home renovation [47]. Based on the question of whether there is a home renovation within the last 1 year, including adding furniture and painting walls, 19.0% of the 383 school-aged children were found to be from the families whose existing homes had been renovated in the past year. We observed significant discrepancies in the prevalence of impairment in lung function between children without and with home renovation by a chi-square test (Table S5). A statistically significant higher prevalence of impaired lung function was found in the home renovation group compared to those in the non-renovation group, particularly for impaired FEV_6_ (60.6% vs. 42.6%, respectively) and VC (40.8% vs. 26.4%, respectively). After controlling for confounders, though no association was found between house renovation and lower lung function values in our previous study [21], living in renovated houses was associated with a greater risk of lung function impairment in children. It is reported that materials used in home renovation for covering walls, painting, and other furnishings discharge inorganic, organic, and other particles issuing large contents of VOCs and particulate matter into indoor environments [49]. Airborne VOCs exposure is associated with short-term changes in cardiovascular and respiratory function [48]; and particulate exposure has a causal role in the impaired lung function [16]. Thus, though we could not identify the indoor air pollution in renovated houses, we provided new evidence suggesting that home renovation could have an impact on children’s impaired lung function.

In this study, we observed 8.6% of children were born preterm among 383 participants; preterm birth was not statistically associated with lower lung function as we previously reported [21]. However, we found an OR of 4.133 (95% CI: 1.531, 11.158) for impaired FEV_6_, an OR of 3.370 (95% CI: 1.244, 9.131) for impaired FEV_3_, and an OR of 9.518 (95% CI: 1.102, 82.197) for impaired FIVC, respectively, to preterm birth. The findings are supported by the increasing evidence which indicated a reduced and impaired lung function in premature infants, including late preterm infants [50,51]. One possible interpretation for the impairment in lung function caused by preterm birth is that children born prematurely are at high risk of airway obstructions and reduction in small airways patency at various school ages due to multi-factors [52], which can lead to an immature lung and consequently make the lung sensitive to poor early life exposures [53]. Preterm birth itself, combined with these injuries, can lead to impaired lung growth [54]. In line with the study conducted in Lanzhou [42], which indicated that stunting was significantly associated with lower lung function among 6–13 years children 25 years ago, we observed a significant association between impaired ERV and height stunting (OR = 21.170, *p* = 0.043), although a low prevalence of height stunting (1.0%) was found. 

The effects of physical exercise on impaired lung function in children remains at a lack of consensus. The beneficial effects of physical exercise have been informed in children based on a cohort study in China [15], as well as in a prospective study in Europe which also indicated that physical exercise was associated with high lung function [55]. However, some scholars reported that daily physical exercise did not cause long term improvements in lung function [56]. In this study, the deficit in impaired FEV_6_ was observed (OR = 2.265, 95% CI: 1.058, 4.845) for the children doing physical exercise longer than 30 min/day, which is contrary to the results of studies conducted in other cities of China at the same time [17]. Physical exercise is known to improve muscle strength and physical ability, however, the underlying pathophysiology or mechanics behind the effect of physical exercise on lung function is not clear. The different definitions and categorizations of physical exercise, as well as the measurement of physical exercise, etc., may lead to this discrepancy. We tried to assess the associations between sweat conditions during physical exercise, the frequency of doing physical exercise, the type of physical exercise, and the prevalence of impaired lung function, but seldom significant results were found. Thus, more accurate measurements of physical exercise on the type, intensity, and duration, etc., should be further conducted to assess the effects of physical exercise on children’s lung function.

Our contemporaneous study conducted in Chongqing indicated that the use of air conditioning for more than 8 h/day in summer had a detrimental effect on asthma, allergic rhinitis, and bronchitis, while, ventilation for less than 12 h/day during summer was a risk factor for allergic rhinitis [17]. In this study, we observed that opening windows longer in summer and winter were protecting factors for impaired lung function, while, opening windows longer in autumn was a risk factor after adjusting for confounders. However, using air conditioning for a longer time in summer was found to have a negative effect on the impaired lung function, while, using air conditioning longer in autumn had a positive effect on the development of children’s lung function. To ascertain the health effects of window opening time and air conditioner use time in different seasons on the impairment in lung function, we divided the time into two levels to get a dichotomous variable for each season, as shown in Table S6. It shows that opening windows more than 14 h/day in summer and more than 9 h/day in winter decreased the risk for impaired FEF_25_ and FEF_50_, respectively. On the other hand, opening windows for more than 14 h/day in autumn increased the risk of impaired IC, using air conditioning more than 8 h/day in autumn was a benefit factor for impaired FIVC and ERV. Since there was no significant difference in the concentration of 6 main pollutants among the four seasons (Figure S2), which avoided the impact of outdoor air pollution on household indoor air quality caused by opening windows, these results imply that household air exchange in summer and winter, and cool household conditions in autumn would be good for the development of children’s lung function.

We used parental occupation and parental education level to indicate socioeconomic levels. The father being white collar and with a higher education level showed a protective effect on the development of children’s lung function, but, the mother being white collar was found to be associated with higher risk of impaired lung function. To uncover information hidden beneath occupational stratification, we analyzed the distribution characteristics of preterm birth, home renovation, doing physical exercise more than 30 min/day, window opening times in different seasons, and air conditioning use time in summer by different maternal occupations, as shown in Table S7. We found that more children whose mothers were white collar workers came from the families opening windows less than 9 h/day in winter (49.5%, *p* = 0,008), and from the families using air conditioning more than 8 h/day in summer (57.6%, *p* = 0.001), in comparison with other children whose mother occupied other occupations. Additionally, the children whose mother were white collar workers were more likely born preterm, and were mainly from the families having risk factors to impairment in lung function, including household renovation, opening windows less than 14 h/day in summer and opening windows more than 14 h/day in autumn, and doing physical exercise more than 30 min/day, although no statistical difference was observed. These results indicate that other living habits, such as using air conditioning more than 8 h/day in summer and opening windows less than 9 h/day in winter, rather than a higher socioeconomic level were the determinants of impaired lung function in children. 

This study has several notable limitations. The predominant risk factors of home renovation to the impairment of children’s lung function cannot been detailed in totality, since we didn’t survey the main materials used in renovations. As we paid no attention to the original decoration materials of the house, which may have a long impact on children’s lung function, it may consequently bring some uncertainties to the association between home renovation and lung function. Secondly, the impact of some factors on children’s lung function was complex, such as the detrimental health effect of a rural living environment on the impaired lung function. We did not measure the actual environmental pollution in rural and urban areas, as well as in traffic environment, such as air pollution during commuting to school, thus the study was limited in providing any ascertained evidence in relationship between impaired lung function and living environmental exposure and air pollutant exposure during commuting. Thirdly, a relatively small number of participants were recruited to take part in lung function measurements, therefore it is possible that the results were more representative to the particular study population and specific location where the study was conducted. Finally, the questionnaire was self-reported, and we were not in a position to confirm the accuracy of the responses.

## 5. Conclusions

Several previously established beneficial factors of lung function development were ascertained in the current study, such as breastfeeding and higher socioeconomic levels. We further identified the following risk factors that were related to the impairment of children’s lung function, including living in a rural area, preterm birth, and house renovation. As school children living in Wuhan with a subtropical monsoon (humid) climate, we confirmed that opening windows for a longer period of time in autumn and air conditioning use for a longer time in summer would increase the risk of impaired lung function of children. Additionally, the relationship between physical exercise, air pollution exposure, and impaired lung function needs further studies to supplement the existing research.

## Figures and Tables

**Table 1:** Anthropometry, sociodemographic, household, and parental information, and children’s behavior patterns.

Category	Variables	Total (*n* = 383)	Category	Variables	Total (*n* = 383)
Anthropometry	Age, years	9.4 ± 1.2	Socioeconomic factor	Sleep in own room, n (%)	241 (64.8%)
	Male, n (%)	219 (57.2%)		Sleep in own bed, n (%)	288 (77.2%)
	Height, cm	140.6 ± 9.5		Paternal occupation	
	Weight, kg	34.9 ± 9.7		Blue collar ^1^	127 (35.9%)
	BMI status			White collar ^2^	156 (44.1%)
	Normal	283 (74.1%)		Others	71 (20.1%)
	Over weight	53 (13.9%)		Maternal occupation	
	Obese	46 (12.0%)		Blue collar ^1^	90 (26.1%)
	BMI (kg/m^2^)	17.4 ± 3.1		White collar ^2^	193 (55.9%)
	Height stunting, n (%)	4 (1.0%)		Others	62 (18.0%)
	Breast feeding, n (%)	292 (81.1%)		Father education level, ≥College, n (%)	133 (36.0%)
	Preterm birth, n (%)	33 (8.6%)		Mother education level ≥ College, n (%)	120 (32.4%)
	Urban, n (%)	243 (63.4%)			
Personal activity pattern	Active in physical exercise, n(%)	271 (73.2%)	Household factors	Household coal use, n (%)	11 (2.9%)
	ETS exposure, n (%)	318 (83.0%)		Ventilation use for cooking, n (%)	345 (98.9%)
	Vegetables consumption, n (%)			Enclosed kitchen, n (%)	249 (67.7%)
	0–3 times/month	5 (1.3%)		Home renovation, n (%)	71 (19.0%)
	1–6 times/week	41 (11.1%)		Presence of pets, n (%)	63 (16.7%)
	1–3 times/day	325 (87.6%)		Presence of mold, n (%)	43 (11.3%)
	Fruit consumption, n (%)			Mosquito coil use, n (%)	262 (69.5%)
	0–3 times/month	13 (3.5%)		Air fresheners use, n (%)	56 (14.9%)
	1–6 times/week	59 (15.9%)		Air purifier use, n (%)	79 (21.0%)
	1–3 times/day	299 (80.6%)		Window opening time in spring ^3^	14.7 ± 7.7
	Dairy consumption, n (%)			Window opening time in summer ^3^	14.3 ± 7.4
	0–3 times/month	18 (4.9%)		Window opening time in autumn ^3^	13.9 ± 7.8
	1–6 times/week	48 (12.9%)		Window opening time in winter ^3^	9.2 ± 8.0
	1–3 times/day	305 (82.2%)		Air condition use time in spring ^3^	0.7 ± 2.4
	Seafood consumption, n (%)			Air condition use time in summer ^3^	8.6 ± 5.0
	0–3 times/month	124 (33.4%)		Air condition use time in autumn ^3^	0.6 ± 2.2
	1–6 times/week	201 (54.2%)		Air condition use time in winter ^3^	2.1 ± 3.8
	1–3 times/day	46 (12.4%)	Parental factors	Paternal smoking, n (%)	173 (47.4%)
	High-calorie food consumption, n (%)			Maternal smoking, n (%)	0 (0)
	0–3 times/month	259 (69.8%)		Paternal asthma, n (%)	1 (0.3%)
	1–6 times/week	103 (27.8%)		Maternal asthma, n (%)	0 (0)
	1–3 times/day	9 (2.4%)		Paternal bronchitis, n (%)	19 (5.2%)
	Air pollution exposure during commuting, n (%)	295 (77.0%)		Maternal bronchitis, n (%)	22 (6.1%)

Values are mean (SD) or number (percentage %). ^1^ “White collar” includes teacher, businessperson, clerk, housewife (few cases), and other non-manual laborer occupations. ^2^ “Blue collar” refers to factory worker, construction worker, building cleaning worker, farmer, and other manual laborer occupations. ^3^ The time is counted in hour/day.

**Table 2:** Children’s lung function by sex and residential location.

	Mean (SD) (*n* = 383)	Living Urban (*n* = 243)	Living Suburban (*n* = 140)	*p*-Value	(Urban—Suburban) Difference ^1^ Estimate (95% CI)	Boy (*n* = 219)	Girl (*n* = 164)	*p*-Value
FET, sec	1.3 (0.6)	1.4 ± 0.6	1.1 ± 0.3	<0.0001	0.25 (0.05, 0.44) *	1.3 ± 0.6	1.2 ± 0.5	0.002
FEV_6_, L	1.8 (0.5)	1.9 ± 0.6	1.6 ± 0.3	<0.0001	0.10 (−0.04, 0.25)	1.9 ± 0.6	1.8 ± 0.4	0.052
FEV_3_, L	1.8 (0.5)	1.9 ± 0.6	1.6 ± 0.3	<0.0001	0.12 (−0.02, 0.26)	1.9 ± 0.6	1.8 ± 0.4	0.082
FEF_25_, L/sec	3.2 (0.9)	3.3 ± 1.0	3.0 ± 0.8	0.028	0.04 (−0.21, 0.30)	3.2 ± 0.9	3.1 ± 1.0	0.075
FEF_50_, L/sec	2.5 (0.7)	2.5 ± 0.8	2.4 ± 0.6	0.041	−0.06 (−0.26, 0.14)	2.5 ± 0.7	2.5 ± 0.8	0.678
FIVC, L	1.3 (0.6)	1.4 ± 0.6	1.1 ± 0.5	<0.0001	0.10 (−0.07, 0.26)	1.3 ± 0.6	1.3 ± 0.5	0.682
FIV_1_, L	1.2 (0.5)	1.3 ± 0.5	1.1 ± 0.5	0.003	0.01 (−0.14, 0.17)	1.2 ± 0.6	1.2 ± 0.5	0.629
FIV_1_/FIVC, %	79.5 (28.1)	80.9 ± 25.1	77.0 ± 32.7	0.014	0.03 (−8.91, 8.98)	78.3 ± 30.3	81.0 ± 24.9	0.733
FEV_1_/VC, %	82.9 (21.2)	80.3 ± 19.7	87.5 ± 23.0	0.004	−13.70 (−20.72, −6.69) *	83.8 ± 21.2	81.8 ± 21.2	0.749
FEV_3_/FVC, %	99.7 (3.7)	99.7 ± 3.4	99.6 ± 4.2	1	1.15 (−0.39, 2.68)	99.9 ± 1.6	99.4 ± 5.4	1.000
EVC, L	1.9 (1.2)	2.1 ± 1.2	1.4 ± 1.1	<0.0001	0.75 (0.35, 1.14) *	1.8 ± 1.3	1.9 ± 1.2	0.317
IC, L	1.5 (0.9)	1.6 ± 1.0	1.3 ± 0.7	<0.0001	0.32 (0.02, 0.62) *	1.5 ± 1.0	1.5 ± 0.8	0.833
VC, L	2.2 (0.9)	2.3 ± 1.0	1.9 ± 0.7	<0.0001	0.35 (0.04, 0.65) *	2.2 ± 0.9	2.2 ± 0.9	0.789
ERV, L	0.4 (0.4)	0.4 ± 0.4	0.4 ± 0.5	0.036	0.07 (−0.08, 0.22)	0.4 ± 0.4	0.4 ± 0.5	0.116
MVV, L/min	41.0 (13.8)	40.9 ± 14.9	41.1 ± 11.7	0.459	−1.03 (−5.26, 3.19)	41.2 ± 14.2	40.8 ± 13.3	0.238

* indicates the statistically significant difference, at a *p* value of 0.05. ^1^ Adjusted for age, sex, height, weight, father smoking, mother smoking, sleeping in their own room, sleeping in their own bed, household coal use, ventilation use when cooking, paternal occupation, maternal occupation, paternal education level, maternal education level, breastfeeding, paternal asthma, maternal asthma, paternal bronchitis, and maternal bronchitis. Italic numbers were statistically significant.

**Table 3:** Prevalence of lung function impairment in children with different areas, and the odds ratios (OR) and 95% confidence interval (95% CI) of the prevalence of lung function impairment in children in urban areas versus rural areas.

Index	Prevalence, N (%)					Odd Ratios (95% CI) and *p* Value	
	Total (*n* = 383)	Living Urban (N = 243)	Living Suburban (N = 140)	*p*-Value		OR (95% CI) ^1^	*p*-Value
FET	381 (99.5%)	241 (99.2%)	139 (100%)	0.284		/	/
FEV_6_	179 (46.7%)	103 (42.4%)	76 (54.7%)	0.021		0.574 (0.188, 0.971)	0.042 #
FEV_3_	133 (34.7%)	66 (27.2%)	67 (48.2%)	<0.001		0.454 (0.129, 0.767)	0.011 #
FEF_25_	231 (60.3%)	145 (59.7%)	86 (61.9%)	0.672		0.738 (0.305, 1.785)	0.500
FEF_50_	187 (48.8%)	120 (49.4%)	67 (48.2%)	0.824		1.773 (0.762, 4.122)	0.184
FIVC	315 (82.2%)	196 (80.7%)	119 (85.6%)	0.221		1.367 (0.424, 4.401)	0.601
FIV_1_	291 (76.0%)	182 (74.9%)	109 (78.4%)	0.437		0.834 (0.301, 2.311)	0.727
FIV_1_/FIVC	105 (27.4%)	60 (24.7%)	45 (32.4%)	0.106		0.757 (0.282, 2.028)	0.580
FEV_1_/VC	104 (27.2%)	73 (30.0%)	31 (22.3%)	0.102		2.179 (0.793, 5.984)	0.131
FEV_3_/FVC	2 (0.5%)	1 (0.4%)	1 (0.7%)	0.688		/	/
EVC	141 (36.8%)	72 (29.6%)	69 (49.6%)	<0.001		0.263 (0.109, 0.638)	0.003 #
IC	108 (28.2%)	60 (24.7%)	48 (34.5%)	0.04		0.772 (0.322, 1.855)	0.563
VC	113 (29.5%)	59 (24.3%)	54 (38.8%)	0.003		0.351 (0.142, 0.872)	0.024 #
ERV	289 (75.5%)	182 (74.9%)	106 (76.3%)	0.766		0.582 (0.206, 1.648)	0.308
MVV	365 (95.3%)	232 (95.5%)	132 (95.0%)	0.821		0.704 (0.055, 8.989)	0.787

^#^: statistically significant. /: no data. ^1^ Adjusted for gender, age, height, premature birth, parental education level, parental occupation, father smoking, other smoking, sleeping in their own room, sleeping in their own bed, household coal use, ventilation use when cooking, breast feeding, window opening and air condition use in different seasons, home renovation, presence of pets, presence of mold, mosquito coin use, air freshener use, air purifier use, kitchen type, parental bronchitis, intake of fruit, dairy, seafood and high-calorie food, doing physical exercise, ETS exposure, and air exposure during commuting.

**Table 4:** Adjusted odds ratios (OR) of impaired lung function in relation to socioeconomic, personal behavior pattern, parental, and household factors in children ^1^.

Variables	FEV_6_	FEV_3_	FEF_25_	FEF_50_	FIVC	FIV_1_	FIV_1_/ FIVC	FEV_1_/VC	EVC	IC	VC	ERV	MVV
Age (yr)	0.964	0.872	0.979	0.938	0.765	0.711	1.021	0.493 **	1.072	0.875	1.066	2.271 **	1.481
Height (cm)	1.086 *	1.126 **	1.068	1.038	1.110 *	1.180 **	1.070	1.026	1.035	0.965	1.050	0.978	1.133
Weight (kg)	0.932 *	0.905 **	0.947	0.972	0.911 *	0.896 **	0.934	1.004	0.984	1.006	0.958	1.004	0.947
BMI (kg/m^2^)	1.090	1.091	1.079	1.043	1.153 *	1.092	1.071	0.998	1.008	0.996	1.028	1.012	1.082
District (ref: rural)	0.574 *	0.454 *	0.738	1.773	1.367	0.834	0.757	2.179	0.263 **	0.772	0.351 *	0.582	0.704
Sex (ref: female)	1.086	1.162	0.818	0.741	1.273	1.955	1.019	0.786	1.224	1.344	0.983	1.651	1.017
Breast feeding < 4 month (ref: no)	0.859	0.482	0.736	0.736	0.432	0.197 *	0.490	0.670	0.553	0.702	0.638	1.291	0.089
Breast feeding 4~6 month (ref: no)	0.988	0.552	0.914	0.690	0.156 *	0.165 *	0.741	0.066 **	3.942 *	2.280	2.301	2.762	0.817
Breast feeding > 6 month (ref: no)	1.258	1.185	0.921	0.668	0.258	0.313	1.277	0.876	0.863	0.968	0.837	1.030	0.277
Open window in summer (hour/d)	0.935	1.007	0.905 *	1.010	0.978	1.001	1.042	0.941	0.989	0.973	0.978	1.035	0.645 *
Open window in autumn (hour/d)	1.111 *	1.050	1.050	0.960	1.054	1.004	0.900	1.249 *	1.049	1.057	1.062	0.934	/
Open window in winter (hour/d)	0.908 *	0.966	0.955	0.994	0.940	0.999	1.072	0.933	1.025	0.973	0.968	1.118 *	1.354
Use air condition in summer (hour/d)	1.007	0.973	1.130 *	1.112 *	1.116	1.049	0.937	0.983	0.986	0.994	1.006	0.979	/
Use air condition in autumn (hour/d)	0.998	0.995	0.779	1.084	0.718 *	0.871	1.105	1.086	0.876	1.116	0.885	0.693 *	/
Use air condition in winter (hour/d)	0.978	0.991	0.919	0.910	0.940	1.024	1.029	0.949	1.048	0.943	1.001	1.103	2.274
Home renovation (ref: no)	2.786 **	1.527	1.565	0.814	1.087	1.542	0.553	0.682	1.223	0.800	1.900	1.936	17.195
Presence of pets (ref: no)	1.123	1.228	0.939	1.875	1.566	2.139	2.227	0.760	1.108	1.253	1.461	0.782	/
Presence of mold (ref: no)	0.684	0.616	0.544	0.391	0.533	0.960	0.342	0.421	0.847	1.500	1.010	3.512	0.744
Mosquito coil use (ref: no)	1.085	0.834	1.429	1.038	1.480	0.603	0.258 **	0.667	0.785	1.023	0.880	0.870	2.424
Air freshener use (ref: no)	0.604	0.618	0.974	1.024	0.733	0.900	1.954	2.018	1.655	2.604 *	1.967	0.840	0.027
Air purifier use (ref: no)	1.457	1.493	1.013	0.753	0.709	1.099	0.630	1.061	0.850	0.892	0.685	0.699	1.599
Enclosed kitchen (ref: no)	1.029	1.393	1.230	0.992	0.787	1.255	0.748	0.778	1.490	1.389	1.933	0.901	9.456
Coal fuel use (ref: no)	1.323	1.959	0.276	0.531	0.539	1.138	2.435	3.035	0.353	0.269	0.606	0.526	/
Ventilation use during cooking (ref: no devices)													
Hood use	0.940	1.320	0.821	0.903	3.425	1.719	0.550	0.509	0.455	0.830	0.451	1.577	
Fan use	0.964	3.552	0.263	0.118	1.662	0.668	0.407	0.100	0.809	1.619	0.271	6.866	
Paternal occupation (ref: no stable income)													
Father being blue collar	0.674	0.654	0.639	0.676	1.560	2.418	1.792	2.218	1.074	0.715	1.281	1.185	0.841
Father being white collar	0.845	1.043	0.300 *	0.403	1.028	1.111	0.877	2.226	1.180	0.364 *	1.563	0.778	0.078
Mother occupation (ref: no stable income)													
Mother being blue collar	1.195	1.691	2.222	2.289	0.767	0.536	0.463	1.026	0.825	1.248	0.634	0.645	23.601
Mother being white collar	0.513	0.758	3.650 *	4.116 **	1.449	0.766	0.507	0.730	0.851	2.131	0.553	0.804	4.187
Paternal education (ref: <College)	0.705	0.921	0.383	0.611	0.471	0.539	0.431	1.802	1.022	0.633	1.234	0.337 *	0.924
Maternal education (ref: <College)	1.195	0.769	1.146	0.702	0.945	0.557	1.549	0.842	0.377	0.698	0.411	1.380	0.016
Sleep in own room (ref: yes)	1.771	1.839	1.292	1.448	1.804	2.242	2.855 *	3.525 **	0.928	1.197	0.661	0.785	0.011
Sleep in own bed (ref: yes)	0.976	0.839	1.528	1.023	0.612	0.594	0.703	0.226 **	1.252	1.329	1.517	1.461	17.928
Paternal bronchitis (ref: no)	0.253	0.511	3.300	0.813	0.934	0.184 *	0.960	2.977	0.334	0.568	0.319	1.066	0.006
Maternal bronchitis (ref: no)	0.785	0.650	0.246	0.324	3.205	3.457	0.413	0.554	2.227	0.991	1.705	1.187	0.041
Whether preterm birth (ref: no)	4.133 **	3.370 *	1.607	2.262	9.518 *	2.927	1.318	1.482	1.447	0.850	2.021	1.074	38.675
Height stunting (ref: no)	1.278	0.126	0.386	0.217	/	/	0.100	0.052	/	/	/	21.170 *	61.050
Intake vegetable: (ref: 0–3 times/month)													
1–6 times/week	0.902	0.845	4.955	9.259	/	/	2.461	/	2.991	0.213	1.479	/	/
1–3 times/day	2.222	1.478	1.195	1.776	/	/	0.882	/	2.124	0.266	0.875	/	/
Intake fruit: (ref: 0–3 times/month)													
1–6 times/week	0.242	0.399	3.685	1.784	0.084	0.100	/	0.781	0.238	0.904	0.158	1.165	/
1–3 times/day	0.126	0.151	12.054 *	3.750	0.260	0.152	/	1.393	0.294	0.880	0.173	0.409	/
Intake dairy (ref: 0–3 times/month)													
1–6 times/ week	0.573	1.012	2.641	0.772	6.900	2.597	0.480	2.224	3.940	3.166	3.255	3.423	/
1–3 times/ day	0.351	2.268	0.880	0.517	3.447	1.903	0.307	1.468	2.931	2.993	3.735	4.872	/
Intake seafood (ref: 0–3 times/ month)													
1–6 times/week	1.125	0.955	1.285	1.881	0.956	0.670	0.557	1.339	0.620	1.106	0.475	0.520	/
1–3 times/day	0.813	0.419	1.551	1.629	1.492	0.559	0.455	3.202	0.960	0.504	0.462	0.390	/
Intake highcalorie food (ref: 0–3 times/month)													
1–6 times/week	0.995	0.860	0.820	1.310	1.016	0.463	0.212	0.583	1.146	1.668	1.381	1.187	/
1–3 times/day	1.115	2.514	0.826	0.935	0.163	0.057 *	0.013	0.834	0.466	0.327	1.254	3.394	/
Doing physical exercise (ref: <30 min /day)	2.265 *	1.892	1.798	1.789	2.463	2.163	1.344	1.807	0.791	0.837	0.924	1.858	/
ETS exposure (ref: no)	1.293	2.050	1.307	1.027	0.513	0.678	0.736	2.198	1.818	1.511	1.823	0.537	0.975
Air pollution exposure duringcommuting (ref: no)	2.113	2.195	0.522	1.022	1.524	1.024	1.050	0.745	1.598	0.797	1.751	1.717	0.140

^1^ Since no valid OR values were obtained for FET and FEV_3_/FVC, the ORs were not shown in the table. /: no data since the effect was too low. *: *p* <0.05; **, *p* < 0.01.

## Data Availability

The data presented in this study are available on request from the corresponding author. The data are not publicly available due to privacy restrictions.

## Supplementary Materials

The following supporting information can be downloaded at: https://www.mdpi.com/article/10.3390/ijerph20021134/s1, Figure S1: Introduction of different kitchen styles; Figure S2: Concentrations of six main pollutants in different seasons of Wuhan, China; Table S1: Anthropometry, sociodemographic, household and parental information and children’s behavior patterns by residence location (urban versus suburban); Table S2: Prevalence of reduced lung function in children with different gender and areas, and the odds ratios (OR) and 95% confidence interval (95% CI) of the prevalence of reduced lung function in children in male versus female; Table S3: Lung function and the prevalence of impaired lung function in children with different BMI status, bold numbers were statistically significant; Table S4: Adjusted odds ratios (OR) of impaired lung function in relation to different BMI in children, bold numbers were statistically significant; Table S5: Prevalence of reduced lung function in children with different potential influence factors; Table S6: Adjusted OR (95% CI) for decreased lung function and opening window, using air condition in different seasons for children from urban and rural areas (*n* = 383); Table S7: Rate of preterm birth, household renovation, doing physical exercise of children among mother groups being different occupations.
