# Supplementary material for: Environmental and Behavioral Factors in Association with Lung Function Impairment in Children Living in Wuhan, China
Source: Int J Environ Res Public Health. 2023 Jan 9;20(2):1134. doi: 10.3390/ijerph20021134 (PMC9858857; doi:10.3390/ijerph20021134)
Supplement: Supplementary file 1 [file ijerph-20-01134-s001.zip › ijerph-2107635-supplementary.pdf]

Table S1. Anthropometry, sociodemographic, household and parental information and children's behavior patterns by residence location (urban versus suburban).

Category	Variables	Total (n=383)	Living in urban (n=243)	Living in suburban (n=140)	P-value
Anthropometry	Age, years	9.4±1.2	9.5±1.5	9.3±0.6	**<0.001**
	Male, n (%)	219 (57.2%)	139 (57.2%)	80 (57.1%)	0.947
	Height, cm	140.6±9.5	142.1±10.9	137.9±5.5	**<0.001**
	Weight, kg	34.9±9.7	36.4±11.0	32.5±6.4	**<0.001**
	BMI status				**0.014**
	Normal	283 (74.1%)	168 (69.1%)	115 (82.7%)	
	Over weight	53 (13.9%)	40 (16.5%)	13 (9.4%)	
	Obese	46 (12.0%)	35 (14.4%)	11 (7.9%)	
	BMI (kg/m^2)	17.4±3.1	17.7±3.4	17.0±2.4	**<0.01**
	Height stunting, n (%)	4 (1.0%)	2 (0.8%)	2 (1.4%)	0.564
	Breast feeding, n (%)	292 (81.1%)	180 (75.9%)	112 (91.1%)	**<0.001**
	Preterm birth, n (%)	33 (8.6%)	26 (10.9%)	7 (5.4%)	0.081
Personal activity pattern	Active in physical exercise, n (%)	271 (73.2%)	185 (79.1%)	86 (63.2%)	**<0.001**
	ETS exposure, n (%)	318 (83.0%)	209 (86.0%)	108 (77.7%)	**0.038**
	Vegetables consumption, n (%)				**0.047**
	0-3 times/month	5 (1.3%)	1 (0.4%)	4 (2.9%)	
	1-6 times/week	41 (11.1%)	22 (9.4 %)	19 (14.0%)	
	1-3times/day	325 (87.6%)	211 (90.2)	113 (83.1%)	
	Fruit consumption, n (%)				0.285
	0-3 times/ month	13 (3.5%)	6 (2.6%)	7 (5.1%)	
	1-6 times/week	59 (15.9%)	34 (14.5%)	24 (17.6%)	
	1-3times/day	299 (80.6%)	194 (82.9%)	105 (77.2%)	
	Dairy consumption, n (%)				**0.007**
	0-3 times/ month	18 (4.9%)	7 (3.0%)	11 (8.1%)	
	1-6 times/week	48 (12.9%)	24 (10.3%)	24 (17.6%)	
	1-3times/day	305 (82.2%)	203 (86.8%)	101 (74.3%)	
	Seafood consumption, n (%)				**0.001**
	0-3 times/ month	124 (33.4%)	70 (29.9%)	54 (39.7%)	
	1-6 times/week	201 (54.2%)	143 (61.1%)	58 (42.6%)	
	1-3times/day	46 (12.4%)	21 (9.0%)	24 (17.6%)	
	High-calorie food consumption, n (%)				0.878
	0-3 times/ month	259 (69.8%)	163 (69.7%)	95 (69.9%)	
	1-6 times/week	103 (27.8%)	66 (28.2%)	37 (27.2%)	
	1-3times/day	9 (2.4%)	5 (2.1%)	4 (2.9%)	
	Air pollution exposure during commuting, n (%)	295 (77.0%)	181 (74.5%)	114 (82.0%)	**0.042**
Parental factors	Paternal smoking, n (%)	173 (47.4%)	119 (51.3%)	54 (40.9%)	0.147
	Maternal smoking, n (%)	0 (0)	0 (0)	0 (0)	/
	Paternal asthma, n (%)	1 (0.3%)	0 (0)	1 (0.8%)	0.172
	Maternal asthma, n (%)	0 (0)	0 (0)	0 (0)	/
	Paternal bronchitis, n (%)	19 (5.2%)	14 (5.9%)	5 (4.0%)	0.435
	Maternal bronchitis, n (%)	22 (6.1%)	13 (5.4%)	9 (7.3%)	0.491
Household factors	Household coal use, n (%)	11 (2.9%)	4 (1.6%)	7 (5.0%)	0.057
	Ventilation use when cooking, n (%)	345 (98.9%)	223 (99.1%)	122 (98.4%)	0.543
	Enclosed kitchen, n (%)	249 (67.7%)	171 (72.5%)	78 (59.1%)	**0.009**
	Home renovation, n (%)	71 (19.0%)	46 (19.1%)	25 (18.8%)	0.945
	Presence of pets, n (%)	63 (16.7%)	39 (16.1%)	24 (17.8%)	0.678
	Presence of mold, n (%)	43 (11.3%)	28 (11.5%)	15 (11.0%)	0.885
	Mosquito coil use, n (%)	262 (69.5%)	154 (63.9%)	108 (79.4%)	**0.002**

	Air fresheners use, n (%)	56 (14.9%)	32 (13.3%)	24 (17.6%)	0.252
	Air purifier use, n (%)	79 (21.0%)	60 (24.9%)	19 (14.1%)	**0.013**
	Time of window opening in spring [3]	14.7±7.7	14.9±7.8	14.1±7.3	0.375
	Time of window opening in summer [3]	14.3±7.4	13.7±7.4	15.4±7.3	**0.047**
	Time of window opening in autumn [3]	13.9±7.8	14.2±7.8	13.3±7.8	0.318
	Time of window opening in winter [3]	9.2±8.0	9.2±7.1	9.3±9.8	0.959
	Time of air conditioner use in spring [3]	0.7±2.4	0.9±2.8	0.3±1.4	**0.022**
	Time of air conditioner use in summer [3]	8.6±5.0	9.1±4.8	7.3±5.1	**0.002**
	Time of air conditioner use in autumn [3]	0.6±2.2	0.6±2.4	0.4±1.8	0.080
	Time of air conditioner use in winter [3]	2.1±3.8	2.4±3.9	1.3±3.7	**0.015**
Socioeconomic factor	Sleep in own room, n (%)	241 (64.8%)	169 (70.7%)	72 (54.1%)	**0.001**
	Sleep in own bed, n (%)	288 (77.2%)	199 (83.3%)	89 (66.4%)	**<0.001**
	Paternal occupation				**<0.001**
	Blue collar[1]	127 (35.9%)	64 (26.9%)	63 (54.3%)	
	White collar[2]	156 (44.1%)	112 (47.1%)	44 (37.9%)	
	Others	71 (20.1%)	62 (26.1%)	9 (7.8%)	
	Maternal occupation				**<0.001**
	Blue collar [1]	90 (26.1%)	36 (15.3%)	54 (49.5%)	
	White collar [2]	193 (55.9%)	146 (61.9%)	47 (43.1%)	
	Others	62 (18.0%)	54 (22.9%)	8 (7.3%)	
	Paternal education level				**<0.001**
	≥College, n (%)	133 (36.0%)	116 (48.3%)	17 (13.2%)	
	Maternal education level				**<0.001**
	≥ College, n (%)	120 (32.4%)	110 (45.6%)	10 (7.8%)	

Values are mean (SD) or number (percentage %).

[1] "White collar" includes teacher, businessperson, clerk, housewife (few cases), and other non-manual laborer occupations.

[2] "Blue collar" refers to factory worker, construction worker, building cleaning worker, farmer, and other manual laborer occupations.

[3] The time is counted in min/day

Table S2. Prevalence of reduced lung function in children with different gender and areas, and the odds ratios (OR) and 95% confidence interval (95% CI) of the prevalence of reduced lung function in children in male versus female

Index	Prevalence, N(%)				Odd ratios (95% CI) and P value	
	Total	Male	Female	P	OR (95% CI) [1]	P
FET	381 (99.5%)	218(99.5%)	162(99.4%)	0.834	/	/
FEV_6	179 (46.7%)	98(44.7%)	81(49.7%)	0.338	1.086 (0.583, 2.022)	0.796
FEV_3	133 (34.7%)	71(32.4%)	62(38.0%)	0.254	1.162 (0.602, 2.244)	0.655
FEF_{25}	231 (60.3%)	125(57.1%)	106(65.0%)	0.116	0.818 (0.432, 1.551)	0.539
FEF_{50}	187 (48.8%)	99(45.2%)	88(54.0%)	0.089	0.741 (0.404, 1.360)	0.334
FIVC	315 (82.2%)	181(82.6%)	134(82.2%)	0.911	1.273 (0.571, 2.840)	0.555
FIV_1	291 (76.0%)	169(77.2%)	122(74.8%)	0.598	1.995 (0.914, 4.183)	0.084
FIV_1/FIVC	105 (27.4%)	60(27.4%)	45(27.6%)	0.964	1.019 (0.507, 2.045)	0.959
FEV_1/VC	104 (27.2%)	60(27.4%)	44(27.0%)	0.93	0.786 (0.387, 1.596)	0.505
FEV_3/FVC	2 (0.5%)	0(0)	2(1.2%)	0.1	/	/
EVC	141 (36.8%)	85(38.8%)	56(34.4%)	0.372	1.224 (0.639, 2.342)	0.542
IC	108 (28.2%)	67(30.6%)	41(25.2%)	0.243	1.344 (0.692, 2.603)	0.381
VC	113 (29.5%)	67(30.6%)	46(28.2%)	0.615	0.983 (0.502, 1.926)	0.960
ERV	289 (75.5%)	169(77.2%)	119(73.0%)	0.35	1.651 (0.784, 3.475)	0.187
MVV	365 (95.3%)	207(94.5%)	157(96.3%)	0.412	1.017 (0.117, 8,836)	0.988

/, no data

[1], Adjusted for gender, age, height, premature birth, parental education level, parental occupation, father smoking, other smoking, sleeping in own room, sleeping in own bed, household coal use, ventilation use when cooking, breast feeding, window opening and air condition use in different seasons, home renovation, presence of pets, presence of mold, mosquito coin use, air freshener use, air purifier use, kitchen type, parental bronchitis, intake of fruit, dairy, seafood and high-calorie food, doing physical exercise, ETS exposure and air exposure during commuting

Table S3. Lung function and the prevalence of impaired lung function in children with different BMI status, bold numbers were statistically significant

	**Normal (N=283)**	**Overweight (N=53)**	**Obesity (N=47)**	**Overall (N=383)**	***p*-value**
Lung function levels, Mean (SD)					
FET, sec	1.22(0.51)	1.39 (0.72)	1.36 (0.59)	1.26 (0.55)	0.045
FEV_6, L	1.74 (0.42)	1.99 (0.42)	2.18 (0.93)	1.83 (0.53)	**0.000**
FEV_3, L	1.73 (0.43)	1.98 (0.42)	2.18 (0.93)	1.82 (0.52)	**0.000**
FEF_{25}, L/sec	3.06 (0.89)	3.46 (0.98)	3.60 (0.94)	3.18 (0.93)	**0.000**
FEF_{50}, L/sec	2.42 (0.70)	2.67 (0.71)	2.73 (0.78)	2.49 (0.72)	**0.004**
FIVC, L	1.26 (0.55)	1.38 (0.67)	1.57 (0.66)	1.31 (0.59)	**0.002**
FIV_1, L	1.17 (0.52)	1.26 (0.61)	1.46 (0.59)	1.22 (0.55)	**0.003**
FIV_1/FIVC, %	79.32 (27.65)	76.39 (31.53)	83.81 (26.98)	79.50 (28.11)	0.421
FEV_1/VC, %	83.53 (21.48)	82.32 (20.86)	79.84 (20.21)	82.91 (21.20)	0.538
FEV_3/FVC, %	99.57 (4.33)	100.00 (0.00)	100.00 (0.00)	99.68 (3.72)	0.611
EVC, L	1.75 (1.16)	2.19 (1.21)	2.12 (1.51)	1.86 (1.22)	**0.015**
IC, L	1.44 (0.84)	1.68 (0.88)	1.75 (1.13)	1.51 (0.89)	**0.025**
VC, L	2.07 (0.83)	2.39 (0.99)	2.47 (1.19)	2.17 (0.91)	**0.004**
ERV, L	0.39 (0.42)	0.50 (0.50)	0.40 (0.49)	0.40 (0.44)	0.193
MVV, L/min	39.44 (13.12)	42.62 (14.65)	48.67 (14.49)	41.04 (13.82)	**0.000**
Prevalence of impaired lung function, N (%)					
FET	282 (74.2 %)	52 (13.7 %)	46 (12.1 %)	381 (99.5%)	**0.000**
FEV_6	148 (82.7 %)	13 (7.3 %)	18 (10.1 %)	179 (46.7%)	0.201
FEV_3	109 (82.0 %)	13 (9.8 %)	11 (8.3 %)	133 (34.7%)	**0.000**
FEF_{25}	183 (79.2%)	23 (10.0 %)	25 (10.8 %)	231 (60.3%)	**0.000**
FEF_{50}	144 (77.0 %)	20 (10.7 %)	23 (12.3 %)	187 (48.8%)	0.646
FIVC	237 (75.2 %)	41 (13.0 %)	37 (11.7 %)	315 (82.2%)	**0.000**
FIV_1	220 (75.6 %)	40 (13.7 %)	31 (10.7 %)	291 (76.0%)	**0.000**
FIV_1/FIVC	80 (76.2 %)	14 (14.3 %)	10 (9.5 %)	105 (27.4%)	**0.000**
FEV_1/VC	75 (72.1 %)	13 (12.5 %)	16 (15.4 %)	104 (27.2%)	**0.000**
FEV_3/FVC	2 (100 %)	0 (0 %)	0 (0 %)	2 (0.5%)	**0.000**
EVC	114 (80.9 %)	11 (7.8 %)	16 (11.3 %)	141 (36.8%)	**0.000**
IC	84 (77.8 %)	11 (10.2 %)	13 (12.0 %)	108 (28.2%)	**0.000**
VC	93 (82.3 %)	9 (8.0 %)	11 (9.7 %)	113 (29.5%)	**0.000**
ERV	215 (74.7 %)	39 (13.5 %)	34 (11.8 %)	289 (75.5%)	**0.000**
MVV	269 (73.9 %)	50 (13.7 %)	45 (12.4 %)	365 (95.3%)	**0.000**

Table S4. Adjusted odds ratios (OR) of impaired lung function in relation to different BMI in children, bold numbers were statistically significant

BMI types	FEV_6	FEV_3	FEF_{25}	FEF_{50}	FIVC	FIV_1	FIV_1/FIVC	FEV_1/VC	EVC	IC	VC	ERV
Overweight (ref: Normal)												
OR	3.85	4.70	3.78	2.58	5.64	27.29	2.71	0.88	0.88	0.66	1.46	0.63
95%CI	1.18-12.56	1.34-16.51	1.08-13.19	0.82-8.07	1.31-24.28	5.82-127.87	0.65-11.28	0.25-3.15	0.28-2.73	0.20-2.16	0.43-4.91	0.14-2.85
P	**0.03**	**0.02**	**0.04**	0.10	**0.02**	**0.00**	0.17	0.84	0.82	0.49	0.55	0.55
Obesity (ref: Normal)												
OR	0.93	2.72	1.12	1.06	1.46	4.68	2.55	0.54	0.48	0.46	0.48	0.38
95%CI	0.22, 3.92	0.61-12.22	0.25-4.96	0.26-4.22	0.27-7.85	0.88-25.01	0.48-13.53	0.11-2.71	0.11-2.08	0.10-2.07	0.10-2.34	0.07-2.11
P	0.93	0.19	0.88	0.94	0.66	0.07	0.27	0.46	0.32	0.32	0.37	0.27

[1] Since no valid OR values were obtained for FET, FEV_3/FVC and MVV, the ORs were not showed in the table.

Adjusted for gender, age, height, premature birth, parental education level, parental occupation, father smoking, other smoking, sleeping in own room, sleeping in own bed, household coal use, ventilation use when cooking, breast feeding, window opening and air condition use in different seasons, home renovation, presence of pets, presence of mold, mosquito coin use, air freshener use, air purifier use, kitchen type, parental bronchitis, intake of fruit, dairy, seafood and high-calorie food, doing physical exercise, ETS exposure and air exposure during commuting.

Table S5. Prevalence of reduced lung function in children with different potential influence factors

Index	Renovation			Preterm			Air exposure		
	Yes	No	P	Yes	No	P	Yes	No	P
FET	71 (100.0%)	301 (99.3%)		33 (100.0%)	333 (99.4%)		293 (78.1%)	82 (21.9%)	
FEV_6	43 (60.6%)	129 (42.6%)	*	20 (60.6%)	152 (45.4%)		139 (79.9%)	35 (20.1%)	
FEV_3	28 (39.4%)	98 (32.3%)		15 (45.5%)	112 (33.4%)		108 (84.4%)	20 (15.6%)	*
FEF_{25}	49 (69.0%)	177 (58.4%)		20 (60.6%)	202 (60.3%)		176 (77.5%)	51 (22.5%)	
FEF_{50}	34 (47.9%)	149 (49.2%)		19 (57.6%)	160 (47.8%)		146 (79.8%)	37 (20.2%)	
FIVC	59 (83.1%)	248 (81.8%)		30 (90.9%)	274 (81.8%)		245 (79.0%)	65 (21.0%)	
FIV_1	58 (81.7%)	226 (74.6%)		28 (84.8%)	253 (75.5%)		228 (79.7%)	58 (20.3%)	
FIV_1/FIVC	15 (21.1%)	89 (29.4%)		9 (27.3%)	90 (26.9%)		83 (79.8%)	21 (20.2%)	
FEV_1/VC	14 (19.7%)	88 (29.0%)		10 (30.3%)	93 (27.8%)		81 (78.6%)	22 (21.4%)	
FEV_3/FVC	0 (0.0%)	2 (0.7%)		0 (0.0%)	2 (0.65)		2 (100.0%)	0	
EVC	31 (43.7%)	106 (35.0%)		14 (42.4%)	122 (36.4%)		113 (81.9%)	25 (18.1%)	
IC	23 (32.4%)	81 (26.7%)		11 (33.3%)	94 (28.1%)		78 (75.0%)	26 (25.0%)	
VC	29 (40.8%)	80 (26.4%)	*	13 (39.4%)	97 (29.0%)		91 (82.0%)	20 (18.0%)	
ERV	60 (84.5%)	224 (73.9%)		25 (75.8%)	251 (74.9%)		224 (78.9%)	60 (21.1%)	
MVV	69 (97.2%)	287 (94.7%)		31 (93.9%)	320 (95.5%)		280 (78.0%)	79 (22.0%)	

* indicates statistically significant difference with a *P* value <0.05

Table S6. Adjusted OR (95%CI) for decreased lung function and opening window, using air condition in different seasons for children from urban and rural areas (n=383)

Variables	FEV_6	FEV_3	FEF_{25}	FEF_{50}	FIVC	FIV_1	FIV_1/FIVC	FEV_1/VC	EVC	IC	VC	ERV	MVV
Window opening time													
Summer (ref: <14 hours/day)	0.735	1.542	**0.375 ***	0.639	0.817	0.725	0.829	0.724	0.610	0.690	0.415	2.374	/
Autumn (ref: <14 hours/day)	3.043	1.979	1.347	0.888	0.817	1.168	0.407	4.088	2.588	**5.894 ***	4.146	1.193	/
Winter (ref: <9 hours/day)	0.556	1.193	0.622	**0.359 ***	1.671	0.716	1.613	0.608	1.059	1.090	0.861	1.952	/
Air conditioner use time													
Summer (ref: <8 hours/day)	0.600	0.558	2.047	1.261	0.888	0.473	0.681	1.057	0.788	1.558	0.868	1.511	/
Autumn (ref: <8 hours/day)	0.132	0.000	0.030	2.595	**0.010 ****	0.129	5.429	1.695	0.142	4.169	0.125	**0.051 ***	/

Table S7. Rate of preterm birth, household renovation, doing physical exercise of children among mother groups being different occupations

	**Being white collar**	**Being blue collar**	**Without stable income**	***p***
		Household renovation		0.741
No	159 (57.4%)	70 (25.3%)	48 (17.3%)	
**Yes**	**33 (52.4%)**	**17 (27.0)**	**13 (20.6%)**	
		Preterm birth		0.885
No	172 (56.2%)	79 (25.8%)	55 (18.0%)	
**Yes**	**17 (51.5%)**	**9 (27.3%)**	**7 (21.2%)**	
		Doing physical exercise ≥30 min/day		0.118
No	109 (55.6%)	56 (28.6%)	31 (15.8%)	
**Yes**	**56 (57.1%)**	**19 (19.4%)**	**23 (23.5%)**	
		Opening window ≥9 hours/day in winter		0.008
**No**	**104 (49.5%)**	**65 (31.0%)**	**41 (19.5%)**	
Yes	89 (65.9%)	25 (18.5%)	21 (15.6%)	
		Opening window ≥14 hours/day in summer		0.779
**No**	**119 (56.1%)**	**53 (25.0%)**	**40 (18.9%)**	
Yes	74 (55.6%)	37 (27.8%)	22 (16.5%)	
		Opening window ≥14 hours/day in autumn		0.226
No	117 (54.2%)	63 (29.2%)	36 (16.7%)	
**Yes**	**76 (58.9%)**	**27 (20.9%)**	**26 (20.2%)**	
		Using air condition ≥8 hours/day in summer		0.001
No	75 (53.6%)	49 (35.0%)	16 (11.4%)	
**Yes**	**118 (57.6%)**	**41 (20.0%)**	**46 (22.4%)**	

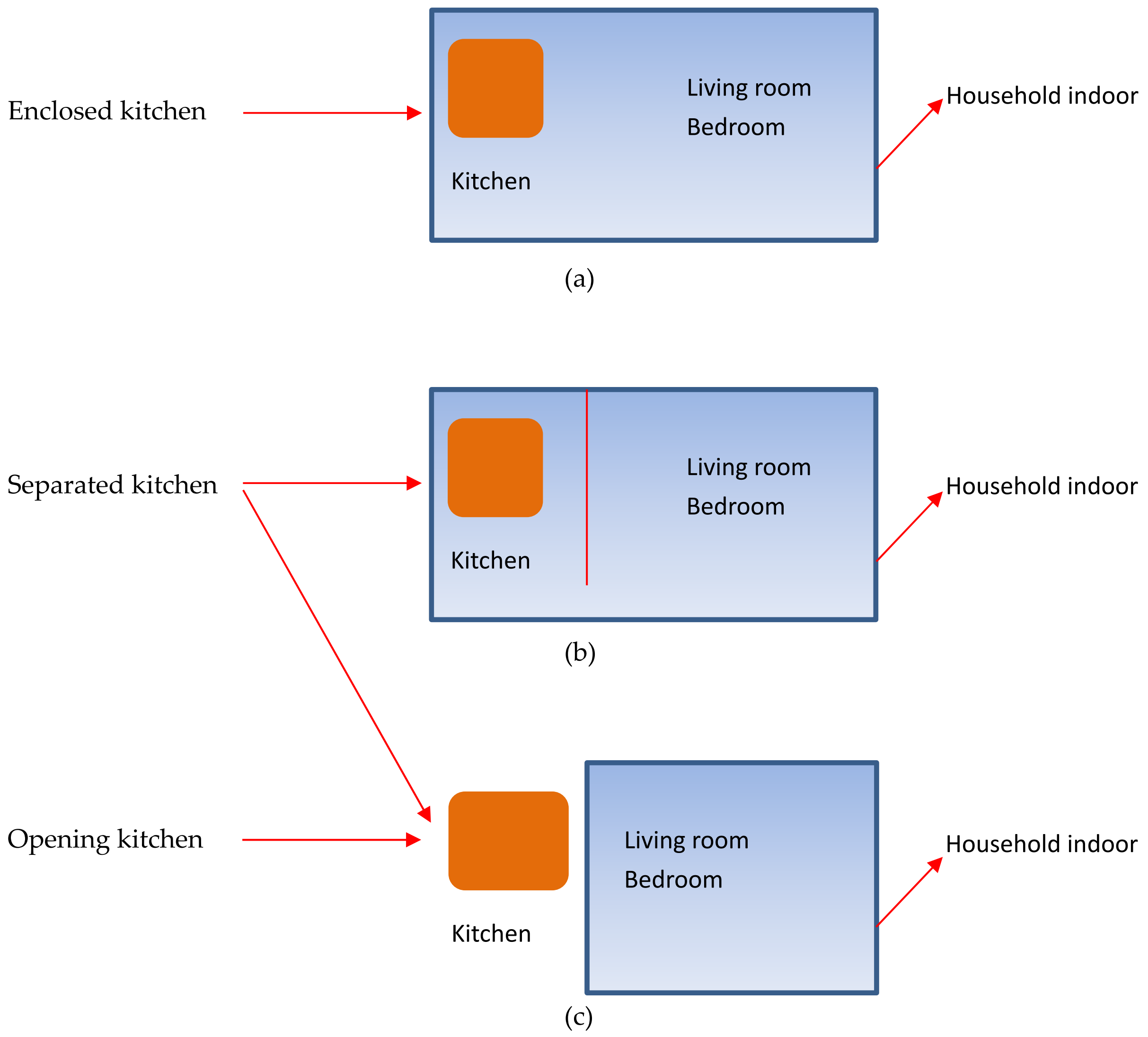

Figure S1. (a): Enclosed kitchen, represents inside or opening kitchen, which is defined as a kitchen that was not isolated to the living room or bedroom with a door or other device; (b): Separated kitchen, represents the kitchen which is isolated to the living room or bedroom with a door or other device, and also represents the kitchen which is separate outside the house (i.e., the kitchen of kind of c); (c): Outdoor kitchen, represents to the kitchen which is separate outside the house

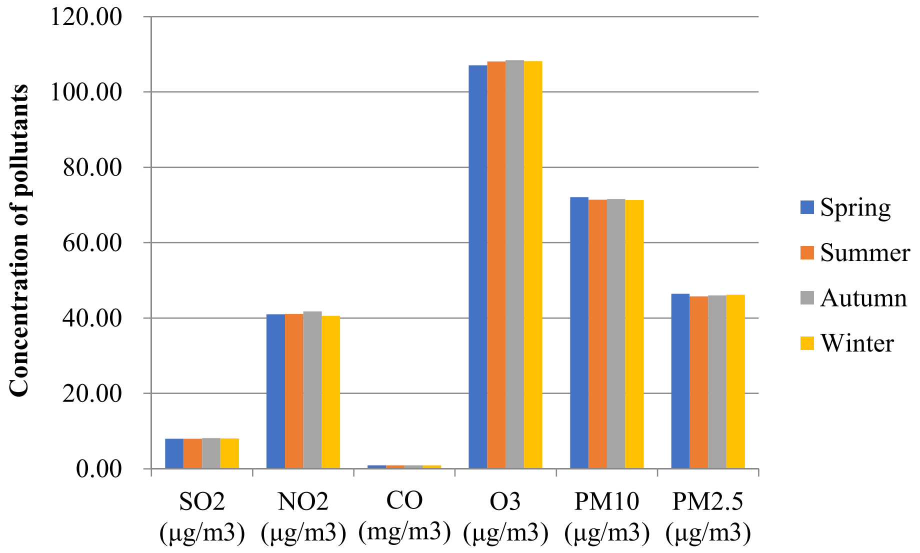

Figure S2. Concentrations of six main pollutants in different seasons of Wuhan, China.
